# Nhe1 is required for directional sensing in vegetative *Dictyostelium* cell migration

**DOI:** 10.1080/19336918.2025.2514374

**Published:** 2025-06-03

**Authors:** Uri Han, Nara Han, Taeck Joong Jeon

**Affiliations:** a Department of Integrative Biological Sciences & BK21 FOUR Educational Research Group for Age-associated Disorder Control Technology, Chosun University, Gwangju, Republic of Korea; b The Basic Science Institute of Chosun University, Chosun University, Gwangju, Republic of Korea

**Keywords:** Cell migration, chemotaxis, *Dictyostelium*, electrotaxis, Nhe1

## Abstract

Na+/H+ exchanger 1 (Nhe1) is a crucial regulator of pH homeostasis and the present study demonstrates that Nhe1 is required for directional sensing in vegetative *Dictyostelium* cell migration. *nhe1* null cells exhibited severe defects in directional sensing and migration in response to folate gradients and applied electric fields.Specifically, *nhe1* null cells failed to exhibit proper translocation of phosphatidylinositol (3,4,5)-trisphosphate (PIP3) to the cell cortex and showed abnormal intracellular calcium signaling upon folate stimulation. Additionally, cells lacking Nhe1 exhibited a reduced capacity to engulf bacteria and latex beads. In contrast, *nhe1* null cells in the developed stage displayed a defect in cAMP-directed chemotaxis, but normal translocation kinetics of PIP3 to the cell cortex upon cAMP stimulation.

## Introduction

Cell migration is a fundamental biological process essential for various physiological functions, including development, immune response, and wound healing. Effective migration requires cells to detect directional cues and translate them into movement, a process known as directional sensing [1,2]. This ability is vital in both unicellular and multicellular organisms. The social amoeba *Dictyostelium* discoideum serves as a powerful model for studying cell migration owing to its well-characterized chemotactic and electrotactic responses [1,2]. Although chemotaxis in aggregation-competent *Dictyostelium* cells in response to cyclic adenosine monophosphate (cAMP) has been extensively studied, little is known about the mechanisms underlying directional migration in vegetative cells, which primarily respond to folate and extracellular electric fields (EFs).

Directional sensing involves the perception of an external signal and establishment of intracellular asymmetry to guide movement. This process is closely linked to cell polarity, which reinforces asymmetry and translates it into the morphological changes necessary for efficient migration. The asymmetric production of phosphatidylinositol (3,4,5)-trisphosphate (PIP3) is a hallmark of directional sensing, while localized actin polymerization, regulated by Rho family GTPases, is a key determinant of cell polarity [1–3]. Actin polymerization drives pseudopod extension and cell movement. The Na+/H+ exchanger (Nhe) family of integral membrane proteins plays a critical role in maintaining the intracellular pH homeostasis by exchanging intracellular H+ with extracellular Na+. Nhe1 is a well-characterized isoform involved in controlling cell morphology, adhesion, motility, and directional migration [4–6]. The intracellular pH balance is essential for numerous physiological processes, including cytoskeletal remodeling, cell proliferation, and apoptosis. In mammalian cells, the disruption of intracellular and extracellular pH regulation contributes to tumorigenesis and disease progression [4,7].

In *Dictyostelium*, intracellular pH plays a crucial role in directed cell migration, as increased H+ efflux facilitates actin cytoskeletal assembly and polarity formation [8]. Nhe1 is predominantly localized at the leading edge of chemotaxing *Dictyostelium* cells, where it regulates the pH-dependent actin polymerization required for migration [8,9]. Previous studies demonstrated that Nhe1 is essential for chemotaxis in developed aggregation-competent cells in response to cAMP, as it contributes to cell polarity by promoting the asymmetric accumulation of cytoskeletal components and phosphoinositides [6,8,10,11]. However, its role in vegetative cell migration, particularly in the response to folate and electric fields, remains unclear. Unlike aggregation-competent cells, which exhibit robust chemotaxis toward cAMP, vegetative *Dictyostelium* cells migrate directionally in response to folate gradients and extracellular EFs [1,12,13]. Electrotaxis or galvanotaxis is a conserved migratory response in many cell types, including epithelial and neural cells, and plays a crucial role in development and wound healing [13,14]. Although the molecular mechanisms of chemotaxis have been extensively studied, the mechanisms by which cells detect and respond to weak direct current electric fields remain poorly understood. Studies suggest that changes in membrane potential, ionic gradients, and cytoskeletal dynamics contribute to electrotactic responses [13–15]; however, the involvement of ion transporters such as Nhe1 in this process is largely unexplored.

In this study, we investigated the role of Nhe1 in the directional sensing and migration of vegetative *Dictyostelium* cells. We demonstrated that Nhe1 was essential for maintaining normal cell morphology, adhesion, and motility. Loss of Nhe1 results in reduced cell spreading and adhesion. Furthermore, vegetative *nhe1* null cells exhibit defects in electrotaxis, displaying decreased migration speed and directionality in response to EF stimulation. Similarly, folate-directed chemotaxis was impaired in *nhe1* null cells, characterized by reduced directional accuracy and migration efficiency. These defects are associated with impaired PIP3 accumulation in the membrane and the disruption of intracellular calcium signaling upon folate stimulation. Our results suggest that Nhe1 is a key regulator of directional migration in vegetative *Dictyostelium* cells and influences both chemotactic and electrotactic responses. This study provides new insights into the mechanisms underlying directional sensing by elucidating the role of Nhe1 in vegetative cell migration.

## Materials and methods

### Strains and materials

*Dictyostelium discoideum* cells wild-type A×2cells (DBS0237914), *nhe1* null cells (DBS0236604), and Nhe1-expressing *nhe1* null cells (DBS0236605) were obtained from the dictyBase stock center (IL, USA). All the cells were cultured axenically in HL5 medium or in association with *Klebsiella aerogenes* at 22°C. Knockout strains and transformants were maintained in 10 μg/mL blasticidin or 10 μg/mL G418. A PIP3 reporter plasmid PH-GFP, 700-bp N-terminal PH domain of CRAC was fused to GFP expression vector [16], was obtained from the *Dictyostelium* stock center. The calcium reporter plasmid pDXA-GCaMP3 was described previously [17]. Red fluorescence latex beads (1.0 µm diameter, L2778) for phagocytosis assay, folate, and cAMP were purchased from Sigma-Aldrich (St. Louis, MO, USA). Stock solutions of folate (100 mm) and cAMP (3 mm) were prepared in dH_2_O, respectively.

### Cell area and adhesion analysis

The cell morphology and size were examined as described previously [18]. Exponentially growing cells on the plates were imaged using a phase-contrast microscope (IX71; Olympus) with a camera (DS-Fil; Nikon). The surface of the cells in the images were outlined and the area of the cells were calculated by using ImageJ (National Institutes of Health).

Cell adhesion assay was performed as described previously [19]. Exponentially growing cells on the plates were washed and resuspended at a density of 2 × 10^7^ cells/ml in 12 mm Na/K phosphate buffer (10 mm KH_2_PO_4_, 2 mm Na_2_HPO_4_, pH 6.1). 100 μL of the cells were placed and attached on the 6-well culture dishes. The cells were photographed and counted for calculating the total cell number. The plates were shaken at 150 rpm for 30 min to detach the cells from the plates and then the medium containing detached cells was removed. The number of attached cells were counted and cell adhesion was presented as a percentage of attached cells compared with total cells.

### Random migration

Random migration using vegetative cells was performed using a slightly modified method previously described [20]. 1–2 drops of fully-grown cells (~5 × 10^6^ cells/mL) were added to 3-cm plates containing 3 mL culture medium and allowed to adhere for 30 min. Subsequently, the cells washed 2 times with development buffer (DB; 5 mm Na₂HPO₄, 5 mm KH₂PO₄, 2 mm MgCl₂, and 1 mm CaCl₂) and incubated for additional 30 min. The random migration was recorded for 30 min using an inverted microscope (IX71; Olympus) with a camera (DS-Fil; Nikon) controlled by the NIS-Elements software (Nikon). Trajectory speed was calculated by dividing the total distance traveled by the cell by the time, using ImageJ software (National Institutes of Health).

### Electrotaxis and chemotaxis analysis

Electrotaxis using vegetative cells starved for 3 h was performed as described previously [12,21]. For experiments with developed aggregation-competent cells, exponentially growing cells washed twice and resuspended at a density of 5 × 10^6^ cells/mL in DB buffer. The cells were pulsed with 30 nM cAMP every 6 min for 6 h [12]. The prepared cells were seeded into an electrotactic chamber for 30 min and the unattached cells were removed by washing with DB buffer. A roof of cover glass was placed on the cells within a trough and sealed with silicone grease. Each chamber was then filled with sufficient DB buffer. EF was applied at 15 V/cm through agar salt bridges. Cell migration was recorded at intervals of 1 min for 1 h using an inverted microscope. Time-lapse recordings of cell migration were analyzed using ImageJ software (NIH). Trajectory speed was calculated as described at random migration assay and directedness was measured as cosine θ, where θ is the angle between the direction of the field and a straight line connecting the start and end positions of the cell.

Chemotaxis experiments using the under-agarose chemotaxis assay were performed with vegetative cells and aggregation-competent cells as described previously with a slight modification [22]. Fully grown vegetative cells on culture plates were resuspended at a density of 5 × 10^6^ cells/mL in SM medium (10 g Difco Bacto-Peptone, 10 g glucose, 1 g yeast extract, 1.9 g KH_2_PO_4_, 0.6 g K_2_HPO_4_, 0.43 g MgSO_4_ in 1 L, pH6.5). 100 μL of cells and 1 mm folate were added into the troughs in 1% agarose chemotaxis chamber, respectively. For cAMP-directed chemotaxis assay, aggregation-competent cells were prepared as in electrotaxis experiments and 150 μM cAMP were used as a chemoattractant in 1% agarose chemotaxis chamber in DB buffer. Cell migration 30 min after adding the chemoattractants into the troughs was recorded and analyzed as in electrotaxis experiments. Directionality is a measure of how straight cells move. Cells moving in a straight line have a directionality of 1.

### Fluorescence image acquisition and analysis

To examine the response of vegetative cells to folate, cells expressing PH-GFP or GCaMP3 cultured with bacteria (*K. aerogenes*) on SM plate were collected and washed 3 times and then resuspended at a density of 5 × 10^6^ cells/mL in DB buffer. To examine the response of the cells to cAMP, developed aggregation-competent cells were prepared by washing vegetative cells twice with DB buffer, resuspending them at a density of 5 × 10^6^ cells/mL, and then pulsing them with 30 nM cAMP at 6-min intervals for 6 h. Vegetative or aggregation-competent cells were plated on glass-bottomed 30-mm plates and allowed to adhere for 30 min. Vegetative cells were uniformly stimulated with 100 µM folate. Aggregation-competent cells were uniformly stimulated with 15 µM cAMP. For uniform stimulation, one-tenth volume of the stock solution (1 mm folate and 150 µM cAMP) was quickly and evenly added to the plates to reach a final concentration.

Fluorescence images were taken at time-lapse intervals of 2 s for 1 min using an inverted microscope (IX71; Olympus, Japan) equipped with a camera (DS-Fi1; Nikon, Japan). The frames were captured using NIS-elements software (Nikon) and analyzed using ImageJ software (NIH). Fluorescence intensities were measured with the ROI manager tool in ImageJ, and relative fluorescence intensities were calculated by dividing the intensity at each time point (Et) by the intensity before stimulation (Eo).

### Phagocytosis assay

Exponentially growing *Dictyostelium* cells were collected and washed with DB buffer and then 5 μl of 2 × 10^6^ cells/mL cells were seeded on SM agar 10-cm plates previously coated with *K. aerogenes*. The bacteria-coated SM agar plates were prepared by adding 200 μl of bacterial culture to the plates and incubating them overnight at 37 °C. The diameter of the plaques formed on the plates at 1 day and 3 days after growing the cells were measured.

Latex bead uptake assay was performed as described previously [23]. Log-phase *Dictyostelium* cells were washed twice with DB buffer and resuspended at a density of 2 × 10^6^ cells/mL. 5 mL of the cells were transferred into 50 mL centrifuge tubes and 5 μl of red fluorescence latex beads (1.0 µm diameter, Sigma-Aldrich) were added to the cell suspension to be 25 beads per cell. The mixture of cells and latex beads was incubated for 30 min with shaking at 150 rpm, and 500 μl of the cells were collected from the tubes at every 5 min, followed by washing twice with DB buffer to remove floating beads. The final cell pellet was resuspended in 100 µL of DB buffer and the cells were plated on glass-bottomed microwell plates and allowed to adhere for 30 min. Fluorescence images were captured using an inverted microscope. The phagocytic efficiency was exhibited as the number of beads ingested by the cells and the percentage of phagocytic cells that ingest the beads at each time point. The number of beads ingested by a cell was calculated by dividing the total number of beads observed inside the cells by the total number of cells, including cells that did not uptake beads. The percentage of phagocytic cells was calculated by dividing the number of cells that ingested at least one bead by the total number of cells.

### Statistical analysis

Statistical analysis was performed using Student’s *t*-test (two-tailed) or one-way ANOVA with a post-hoc Tukey test. All data was collected from at least three independent experiments and expressed as the means ± standard error of the mean (SEM). *p* value less than 0.05 was considered as statistically significant.

## Results

### Nhe1 is involved in controlling cell morphology, adhesion, and random motility

**Figure 1.**
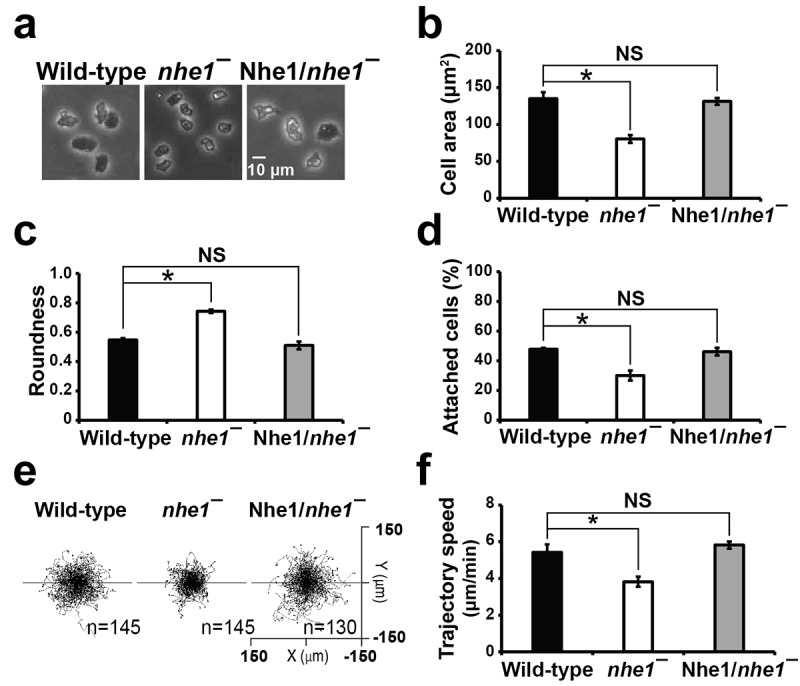
Cell morphology, adhesion, and random motility. (a) morphology of wild-type A×2cells, *nhe1* null cells, and Nhe1-expressing *nhe1* null cells. A×2wild-type cells were used as the parental strain for *nhe1* null cells. Exponentially growing cells on the plate were photographed. Scar bar = 10 μm. (b) quantification of cell area. The outlines of cells in the images were traced, and their areas were calculated using image J software. Values represent the mean ± SEM of three independent experiments. Statistical analysis was performed using one-way ANOVA followed by a post-hoc Tukey HSD test. Statistically different from the control, *p < .05. (c) quantification of roundness. Roundness was calculated by 4*area/(π*major_axis^2). A roundness value closer to 1.0 indicates a more circular shape. As the values approach 0.0, it indicates a more elongated or irregular shape. (d) cell-substrate adhesion. Adhesion was expressed as the percentage of attached cells relative to total number of cells. Values are the mean ± SEM of three independent experiments. (e) cell migration trajectories during random migration. Movements of the cells in random migration were recorded for 30 min at 1-min intervals. Migration paths were tracked using ImageJ and plotted with the start position of each cell centered at (0,0). *N* = 145, 145, and 130 cells for wild-type, *nhe1* null, Nhe1-expressing *nhe1* null cells, respectively. (f) quantification of cell motility during random migration. Trajectories were analyzed using chemotaxis and migration tool in ImageJ. Trajectory speed indicates the speed of cell movement along the total path.

Upon starvation, *Dictyostelium* cells aggregate via cAMP chemotaxis and undergo development to form multicellular fruiting bodies. The role of Nhe1 in developed aggregation-competent cells has been studied in the context of chemoattractant-mediated directional migration, as these cells are sensitive to cAMP stimulation [1,10,11]. In contrast, vegetative cells do not respond chemotactically to cAMP but are responsive to folate and extracellular electric fields. To investigate the role of Nhe1 in vegetative cells, we examined the cell morphology, adhesion, and random motility (Figure 1).

*nhe1* null cells were smaller and more rounded than the wild-type cells (Figure 1a). Measurement of cell areas using ImageJ software showed that *nhe1* null cells were approximately two-thirds the size of wild-type cells (Figure 1b). The roundness of wild-type cells was approximately 0.54, whereas *nhe1* null cells exhibited highly increased roundness of 0.74. This was restored to 0.51 by overexpression of Nhe1 (Figure 1c). Cells lacking Nhe1 showed decreased adhesion (Figure 1d). Wild-type cells showed approximately 50% attachment after the detached cells were washed off by shaking the plate, whereas *nhe1* null cells exhibited only ~ 30% attachment. These defects were rescued by Nhe1 expression, suggesting that Nhe1 plays a role in controlling cell spreading and cell-substrate adhesion in the vegetative state. Nhe1 is involved in F-actin reorganization via a pH-dependent interaction with Aip1 [10]. Random motility analysis using the vegetative cells showed that *nhe1* null cells had a reduced trajectory speed (3.5 μm/min) compared to wild-type cells (5.0 μm/min) (Figure 1e,f).

### Nhe1 is required for EF-directed cell migration in vegetative Dictyostelium cells

**Figure 2.**
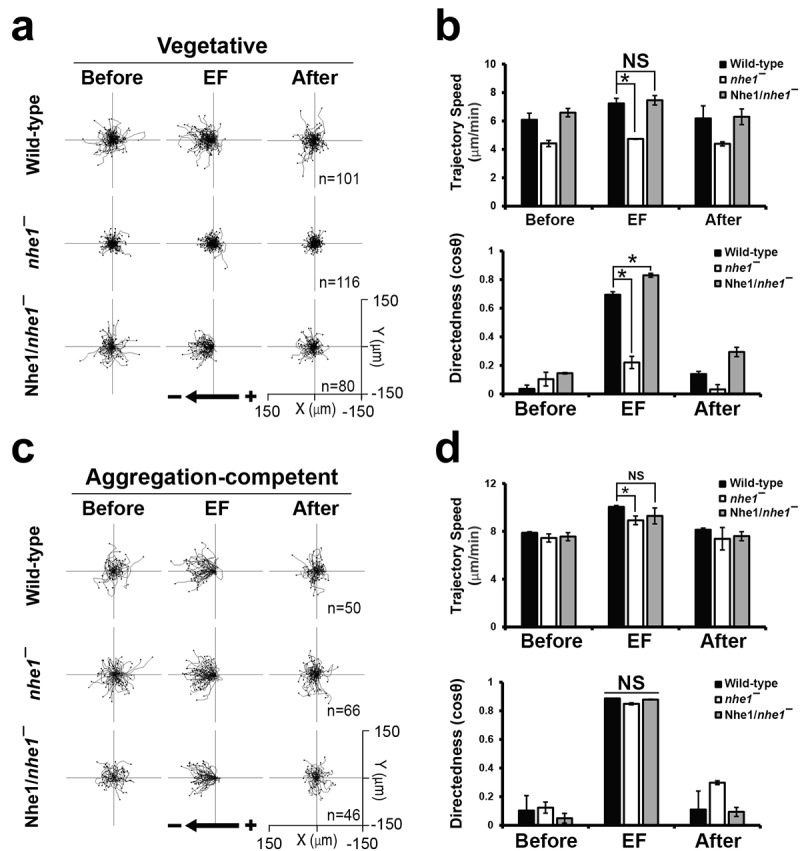
Electrotaxis of vegetative and developed cells. (a) migration trajectories of vegetative wild-type cells, *nhe1* null cells, and Nhe1-expressing *nhe1* null cells during electrotaxis. Cells were exposed to an electric filed (EF) of 15 V/cm, and their movements were recorded at time-lapse intervals of 1 min for 60 min. No EF was applied for the first 10 min and the last 20 min of the experiment. An EF of 15 V/cm was applied between 10 and 40 min. ‘Before’ indicates the first 10 min prior to EF application, ‘EF’ indicates the 10-min period from 20 min to 30 min after EF application, and ‘after’ indicates the 10-min period immediately following EF removal. Cell migration paths were plotted with start positions centered at (0,0). Cells migrated toward the cathode (left). The arrow indicates EF direction. (b) quantitative analysis of directional migration in vegetative cells in an EF of 15 V/cm. Trajectory speed (upper panel) and directedness (lower panel) were compared between cell lines at different time points; 10 min before EF application, 10 min after EF application, and 10 min after EF removal. (c) migration trajectories of developed wild-type cells, *nhe1* null cells, and Nhe1-expressing *nhe1* null cells during electrotaxis. (d) quantitative analysis of the directional migration of developed cells in an EF of 15 V/cm. Data are means ± SEM from three independent experiments. Statistical significance was determined using one-way ANOVA with a post-hoc Tukey test (*p<.05).

Nhe1 functions as a sodium-hydrogen exchanger, affecting pH homeostasis and membrane potential. The intracellular hydrogen ion concentration plays a conserved role in cytoskeletal assembly and cell motility [5,7–9]. *nhe1* null cells exhibited increased intracellular pH and aberrant F-actin assembly [8,9]. To assess the role of Nhe1 in EF-directed migration, we examined directional cell migration in response to EFs using vegetative and developed *Dictyostelium* cells (Figure 2). Cell migration was recorded for 60 min, with no EF applied for the first 10 min, followed by 30 min of 15 V/cm EF application and an additional 20 min without EF. Before EF stimulation, vegetative wild-type cells exhibited random migration (6.0 µm/min of trajectory speed and 0.05 of directedness). Upon EF stimulation, wild-type cells migrated toward the cathode, with increased migration speed (7.1 µm/min) and directedness (0.67). In contrast, vegetative *nhe1* null cells showed significantly lower directedness (0.24) and no increase in trajectory speed after EF application. Nhe1 expression rescued these defects (directedness: 0.79; trajectory speed: 7.2 µm/min; Figure 2a,b).

Electrotaxis experiments using the developed cells revealed no significant difference in directionality among wild-type, *nhe1* null, and Nhe1-expressing *nhe1* null cells, although *nhe1* null cells exhibited a slightly reduced migration speed compared to wild-type cells (Figure 2c,d). All cells in the developed state showed increased migration speed and directionality when EF was turned on, and then decreased to basal levels when EF was turned off. These results suggest that Nhe1 is essential for EF-directed migration in vegetative cells, but not in developed cells.

### Nhe1 is required for folate-directed chemotactic migration in vegetative Dictyostelium cells

**Figure 3.**
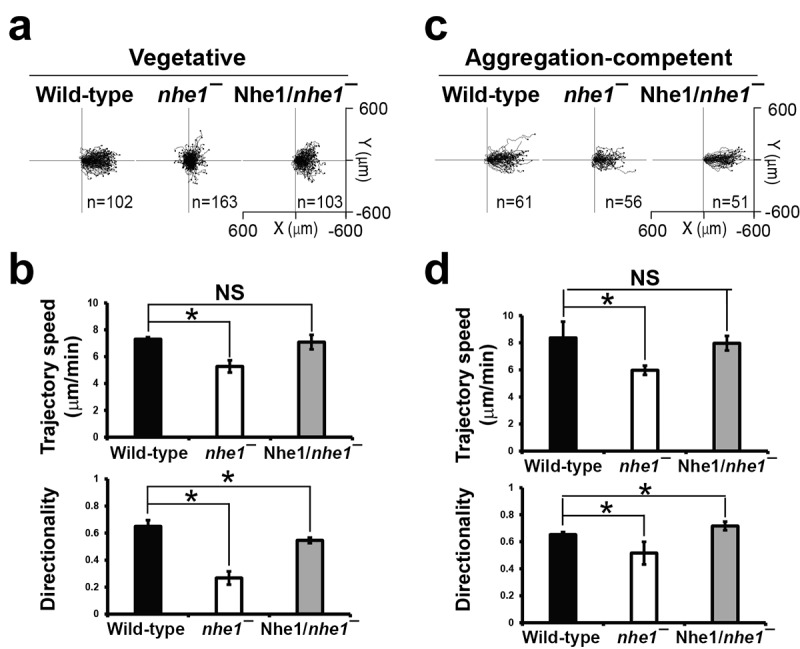
Chemotaxis of vegetative and developed cells. (a) migration trajectories of vegetative cells chemotaxing toward a folate gradient. Wild-type cells, *nhe1* null cells, and Nhe1-expressing *nhe1* null cells were placed in an under-agarose chemotaxis chamber with a folate gradient. Cell movement was recorded at time-lapse intervals of 1 min for 1 h. Migration paths were plotted with the start position centered at (0,0). Cells migrated toward the increasing folate gradient (right). Each line represents the trajectory of a single cell. (b) quantitative analysis of folate-directed chemotaxis in vegetative cells. Trajectory speed indicates the migration speed along the total path, and directionality quantifies how straight the cells move. A directionality value of 1 represents perfectly straight movement. Date are the means ± SEM of three independent experiments. *p < .05 compared to the control (one-way ANOVA with a post-hoc Tukey test). (c) migration trajectories of developed cells chemotaxing toward a cAMP gradient. Cells were placed in a cAMP gradient, and migration was recorded as for folate-directed migration. (d) quantitative analysis of cAMP-directed chemotaxis in developed cells.

Nhe1 has been implicated in the formation of polarity during cAMP-directed chemotaxis in developed aggregation-competent cells [8]. However, their roles in the vegetative cells remain unclear. Here, we examined whether Nhe1 was required for chemotactic migration depending on the cell state (Figure 3).

In the under-agarose assay, vegetative wild-type cells migrated toward folate with an average trajectory speed of 6.8 μm/min and directionality of 0.66. which is a measure of how straight the cells move toward the chemoattractants. In contrast, vegetative *nhe1* null cells showed highly decreased directionality (0.23), almost random migration, and slightly decreased migration speed (4.7 μm/min) compared to wild-type cells (Figure 3a,b). These defects were rescued by Nhe1 expression. In cAMP-directed chemotaxis using developed aggregation-competent cells, *nhe1* null cells exhibited slightly decreased trajectory speed (6.0 µm/min) and directionality (0.51) compared to those in wild-type cells (Figure 3c,d). These findings are consistent with those of previous studies and suggest that Nhe1 plays a critical role in the folate-directed chemotaxis of vegetative cells as well as in the cAMP-directed chemotaxis of aggregation-competent cells.

### Vegetative nhe1 null cells are defective in PIP3 accumulation upon folate stimulation

**Figure 4.**
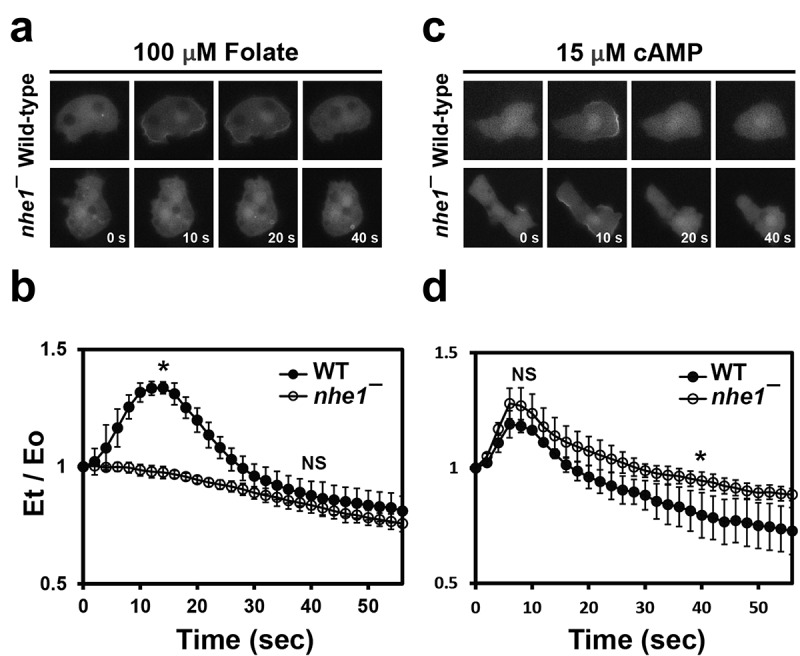
Translocation of PH-GFP to the cell cortex upon stimulation. (a) translocation of PH-GFP to the cell cortex in response to folate stimulation. Vegetative wild-type and *nhe1* null cells expressing PH-GFP were uniformly stimulated with folate. Fluorescence images were captured at 2-s intervals for 1 min. Representative images at 0, 10, 20, and 40 s after stimulation are shown. (b) translocation kinetics of PH-GFP to the cell cortex upon folate stimulation. Fluorescence intensity at the cell cortex was quantified from time-lapse recordings. Graphs show the mean values from multiple cells in three separate experiments. *N* = 59 and 90 cells for wild-type and *nhe1* null cells, respectively. Error bars indicate SEM. Student’s *t*-test at 14 s and 40 s upon folate stimulation. Statistically different from the control, *p < .05. NS, not significant. (c) translocation of PH-GFP to the cell cortex in response to cAMP stimulation. Developed cells expressing PH-GFP were uniformly stimulated with cAMP, and images were captured as described for folate stimulation. Representative images at 0, 10, 20, and 30 s after cAMP stimulation are shown. (d) translocation kinetics of PH-GFP to the cell cortex upon cAMP stimulation. *N* = 46 and 32 cells for wild-type and *nhe1* null cells, respectively. Student’s *t*-test at 10 s and 40 s upon cAMP stimulation. *p < .05. NS, not significant.

Directional cell migration is mediated by a series of signaling molecules, including membrane receptors, Ras proteins, and Pi3K signaling pathways [1,2]. To understand how Nhe1 is involved in regulating directionality during electrotaxis and chemotaxis, we investigated the translocation of PH-GFP, a PIP3 reporter [16], in response to chemoattractant and EF stimuli. It has been well established that cells are polarized and directionally move up to a high concentration of chemoattractants by the asymmetrical localization of PI3K and PTEN. The reciprocal localization of PI3K at the leading edge and PTEN in the posterior region results in PIP3 accumulation at the front, driving cytoskeletal rearrangement for migration [1,2]. We expressed PH-GFP in wild-type and *nhe1* null cells and examined its localization after global chemoattractant or electrical stimulation (Figure 4).

Vegetative wild-type cells showed rapid and transient translocation of PH-GFP to the plasma membrane, peaking at approximately 15 s after uniform global folate stimulation. In contrast, *nhe1* null cells did not exhibit this transient translocation after folate stimulation (Figure 4a,b). Instead, PH-GFP localization remained at a low level on the membrane, with fluorescence intensity remaining constant after stimulation. These data indicate that Nhe1 is required for PH-GFP translocation in response to folate chemoattractant stimulation in vegetative cells.

In developed aggregation-competent cells stimulated with cAMP, both wild-type and *nhe1* null cells showed transient translocation of PH-GFP to the membrane. The translocation kinetics of PH-GFP were similar in the two cell types, with a peak at approximately 10 s, although there was a slight delay in recovery to the basal level in *nhe1* null cells (Figure 4c,d). These results suggest that vegetative *nhe1* null cells have defects in establishing polarity through asymmetric PIP3 accumulation in response to folate stimulation.

When stimulated with EF, neither the wild-type nor *nhe1* null cells exhibited significant PH-GFP translocation from the cytosol to the membrane. Similarly, no changes in the fluorescence intensity were observed in response to high concentrations of KCl, which alters the membrane potential (Fig. S1). These results suggest that distinct mechanisms govern chemotaxis and electrotaxis. Transient translocation of PH-GFP appear to be dispensable for directed cell migration upon EF stimulation.

### Nhe1 is required for intracellular calcium increase upon folate stimulation

Calcium is involved in diverse cellular processes such as cell migration, adhesion, and phagocytosis [7]. To further explore the role of Nhe1 in vegetative cells during migration, we examined calcium dynamics in response to chemoattractant using the *Dictyostelium* specific calcium biomarker GCaMP3 [17].

**Figure 5.**
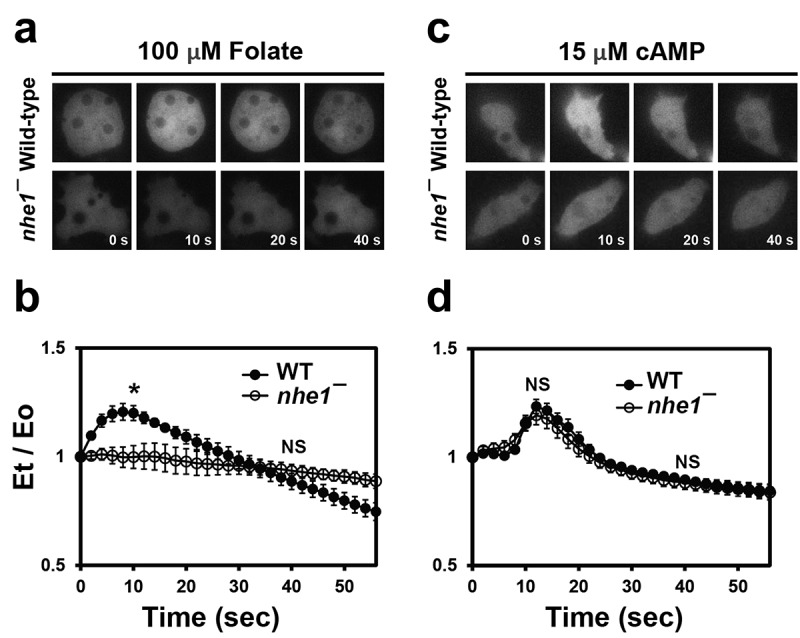
Intracellular calcium response upon chemoattractant stimuli. (a) intracellular calcium response in vegetative cells upon folate stimulation. Vegetative wild-type and *nhe1* null cells expressing GCaMP3 were uniformly stimulated with folate. Fluorescence images were taken with time-lapse intervals of 2 s for 1 min. Representative images at the indicated time points are shown. (b) quantitative analysis of intracellular calcium levels in vegetative cells upon folate stimulation. Fluorescence intensity in the cytosol was measured from time-lapse recordings using ROI Manager in ImageJ. Graphs represent mean values from multiple cells in at least three independent experiments. *N* = 190 and 138 cells for wild-type and *nhe1* null cells, respectively. Error bars indicate SEM. Student’s *t*-test at 10 s and 40 s upon folate stimulation. Statistically different from the control, *p < .05. NS, not significant. (c) intracellular calcium response in developed cells upon cAMP stimulation. Developed cells expressing GCaMP3 were uniformly stimulated with cAMP. (d) quantitative analysis of intracellular calcium levels in developed cells upon cAMP stimulation. Fluorescence intensities were quantified and graphed as described for folate stimulation. *N* = 109 and 189 cells for wild-type and *nhe1* null cells, respectively. Student’s *t*-test at 12 s and 40 s upon cAMP stimulation. *p < .05. NS, not significant.

First, we analyzed intracellular calcium levels in response to folate stimuli in vegetative cells. Wild-type and *nhe1* null cells were exposed to a high folate concentration (final 100 μM) (Figure 5a,b). Wild-type cells expressing GCaMP3 exhibited a transient fluorescence increase in the cytosol following folate stimulation, peaking at approximately 10 s with an intensity ~ 1.3 times the basal level, and then returning to the basal level within 40 s. Interestingly, *nhe1* null cells did not exhibit this increase in fluorescence. Instead, they exhibited a slight initial decrease, returning to the baseline within 40 s (Figure 5b).

Next, we examined the intracellular calcium dynamics in response to cAMP in the developed aggregation-competent cells. Wild-type cells showed a rapid and transient increase in fluorescence upon cAMP stimulation, with a peak at approximately 15 s. *nhe1* null cells displayed slightly lower fluorescence intensity than wild-type cells but exhibited similar kinetics (Figure 5c,d). These results indicate that Nhe1 is crucial for intracellular calcium dynamics in response to chemoattractants, particularly in vegetative cells.

### nhe1 null cells are defective in phagocytosis

*Dictyostelium* cells feed on bacteria via phagocytosis and migrate toward bacteria-secreted folate [24]. Nhe1 is involved in F-actin polymerization by regulating intracellular pH [8,10]. Given the reduced responsiveness of *nhe1* null cells to folate observed in previous experiments, we investigated whether Nhe1 is involved in phagocytosis.

**Figure 6.**
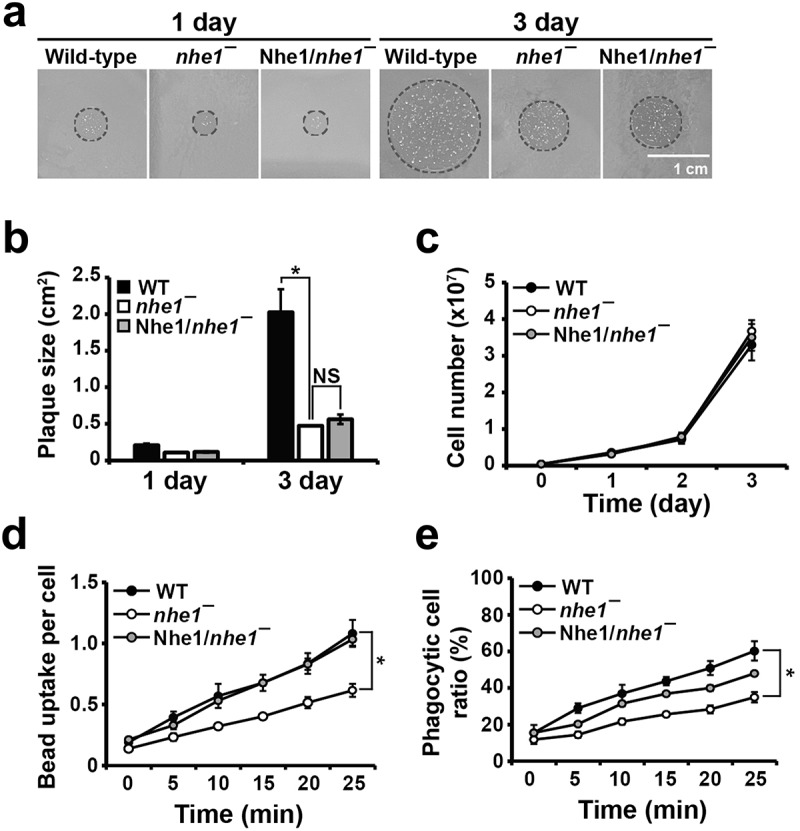
Phagocytosis of bacteria and latex beads. (a) phagocytosis of wild-type and *nhe1* null cells on SM agar plates with *K. aerogenes*. Equal numbers of wild-type and *nhe1* null cells were deposited onto bacterial-coated plates. Cells were grown for 3 days, and the plaques formed by phagocytosis of *Dictyostelium* cells were imaged daily. Representative images at 1 and 3 days are shown. Circles indicate the plaques. (b) quantification of plaque size. Plaque diameters were measured on days 1 and 3 using IamgeJ. (c) growth rates of wild-type, *nhe1* null, and Nhe1-expressing *nhe1* null cells. A total of 4 × 10^6^ cells was grown in shaking culture at room temperature, and cell numbers were measured daily. (d) quantification of bead uptake per cell. The number of beads uptaken was determined by dividing the total number of beads observed inside the cell by the total number of cells, including cells that did not uptake beads. (e) quantification of the percentage of phagocytotic cells. The percentage was calculated by dividing the number of cells that ingested at least one bead by the total number of cells. *p < .05 compared to the control (one-way ANOVA with a post-hoc Tukey test).

Vegetative wild-type and *nhe1* null cells were plated on SM plates with *K. aerogenes* bacteria, and plaque sizes were measured (Figure 6a,b). Three days post-plating, the plaques formed by *nhe1* null cells were significantly smaller than those formed by wild-type cells. However, this phenotype was not restored by expressing Nhe1 in *nhe1* null cells. Further analysis of Nhe1 localization and signaling pathways are required to clarify the underlying cause. The growth rate of *nhe1* null cells in suspension culture was similar to that of wild-type cells (Figure 6c), indicating that the phagocytic defect was not due to impaired cell growth.

To confirm this defect, we performed a phagocytosis assay using latex beads (Figure 6d,e). We quantified the number of beads per cell (Figure 6d) and the percentage of phagocytic cells (Figure 6e). The phagocytic rate was significantly reduced in *nhe1* null cells. After 25 min, *nhe1* null cells contained ~ 0.7 beads per cell, while wild-type cells contained ~ 1.2 beads per cell (Figure 6d). Additionally, 35% of *nhe1* null cells contained beads compared to 60% of wild-type cells at 25 min (Figure 6e). Phagocytic defects were rescued in *nhe1* null cells expressing Nhe1. These results indicate that Nhe1 plays a role in phagocytosis.

### Discussion

The present study highlights the crucial role of Nhe1 in directional sensing and migration of vegetative *Dictyostelium* cells, emphasizing its involvement in folate-directed chemotaxis and electrotaxis. The results indicate that Nhe1 is necessary for maintaining normal cell morphology, adhesion, and motility. Nhe1 is known to contribute to cell migration through multiple mechanisms, including effects on cell volume, intracellular pH regulation, cytoskeletal stabilization, and adhesion [6,8,9,25]. It functions as a key ion exchanger that extrudes H+ in exchange for Na+, thereby maintaining pH homeostasis and preventing intracellular acidification [6]. This process is critical for actin polymerization, as an increase in intracellular pH has been shown to promote the cytoskeletal reorganization necessary for migration. Additionally, Nhe1 acts as a scaffold protein that interacts with actin-binding proteins such as ERM (ezrin, radixin, and moesin) to anchor the cytoskeleton to the plasma membrane, thereby facilitating the formation of lamellipodia at the leading edge of migrating cells [4,6,9].

Our results demonstrate that Nhe1 is essential for electrotaxis in vegetative *Dictyostelium* cells. While wild-type cells showed increased migration speed and directional accuracy upon EF stimulation, while *nhe1* null cells displayed significantly reduced directedness and no enhancement in migration speed. This suggests that Nhe1 plays a key role in sensing and responding to electrical stimuli. Interestingly, the developed aggregation-competent cells lacking Nhe1 exhibited normal directionality and slightly reduced migration speed, indicating that different molecular mechanisms govern electrotaxis in vegetative and developed cells. Similarly, this study reveals a critical role of Nhe1 in the folate-directed chemotaxis of vegetative cells. The *nhe1* null cells exhibited markedly reduced directionality and slower migration speed in response to folate gradients. This defect was accompanied by impaired accumulation of PIP3 at the cell cortex upon uniform folate stimulation, suggesting that Nhe1 regulates polarity formation through PIP3 signaling. In contrast, developed cells lacking Nhe1 were defective in cAMP-directed chemotaxis, but their translocation kinetics of PIP3 to the cell cortex following cAMP stimulation were similar to those of wild-type cells, reinforcing the notion that different regulatory mechanisms are involved depending on the cell state. Studies on mammalian systems have shown that Nhe1-dependent intracellular alkalization promotes cofilin activity, which is necessary for actin polymerization at the leading edge [4,6]. Therefore, the defects observed during folate-directed migration in *nhe1* null cells may occur due to disrupted cofilin regulation and the subsequent failure to remodel the actin cytoskeleton.

These findings are in line with those of previous studies demonstrating that Nhe1-mediated ion transport is required for efficient directional migration in mammalian cells, including fibroblasts, leukocytes, and cancer cells [6,9,25,26]. However, our findings contrast those of previously published studies, which indicate that Nhe1 expression is primarily observed in the developmental stages of *Dictyostelium* rather than in the vegetative state [8]. This discrepancy suggests that Nhe1 May have an unrecognized role in vegetative cell migration, or that its expression levels in the vegetative state are lower but still functionally significant. One possibility is that Nhe1 is expressed at levels below conventional detection thresholds in vegetative cells but remains sufficient to influence chemotaxis and electrotaxis. Further studies, including transcriptomic and proteomic analyses, are required to determine the precise regulation of Nhe1 expression and function in vegetative cells.

Disruption of intracellular calcium dynamics in *nhe1* null cells further elucidates the importance of Nhe1 in directional sensing. It has been previously known that Nhe1 is required for K^+^-facilitated cAMP chemotaxis and Ca^2+^ chemotaxis [11,27]. In this study, wild-type vegetative cells showed a transient increase in cytosolic calcium levels upon folate stimulation, whereas *nhe1* null cells lacked this response. Calcium signaling is crucial for various cellular processes, including actin cytoskeletal remodeling and motility [7], suggesting that loss of Nhe1 disrupts the calcium-mediated signaling pathways necessary for chemotactic migration. The relatively normal calcium response to cAMP in the developed cells further supports the idea that Nhe1 is required for vegetative cell migration. This observation aligns with previous findings in mammalian cells, where calcium influx was shown to be regulated by Nhe1 and required for focal adhesion turnover during migration.

In addition to its role in cell migration, Nhe1 regulates multiple physiological processes, including cell volume control, proliferation, and apoptosis [4,5,7]. In cancer cells, Nhe1 plays a role in tumor progression by enhancing cell motility and invasion through extracellular acidification [9]. Protons extruded by Nhe1 create a low pH microenvironment that facilitates matrix degradation and cell migration [7,9,25]. This suggests that the role of Nhe1 in *Dictyostelium* might extend beyond migration to broader physiological processes, such as phagocytosis and nutrient sensing. Defective phagocytosis observed in *nhe1* null cells provides additional evidence for the involvement of Nhe1 in cytoskeletal regulation. Since *Dictyostelium* cells rely on phagocytosis for nutrient acquisition, the reduced plaque size and bead uptake observed in *nhe1* null cells suggest that Nhe1 is involved in actin-mediated engulfment processes. Interestingly, while Nhe1 expression restored bead uptake, it failed to rescue the reduced plaque expansion (Figure 6). This discrepancy likely reflects the fact that plaque expansion involves not only phagocytosis, but also directional cell migration, chemotaxis toward bacterial signals, and adaptation to complex environmental conditions. Therefore, the inability to restore plaque size may indicate that Nhe1’s role in cell motility and environmental sensing remains partially impaired, even when phagocytic capacity is recovered. Given that phagocytosis and chemotaxis share common signaling pathways, it is likely that the impaired folate-directed chemotaxis observed in *nhe1* null cells results from broader cytoskeletal defects associated with Nhe1 loss. This aligns with the research on Nhe1 in mammalian cells, where it has been shown to regulate cell volume, adhesion, and cytoskeletal rearrangement, thereby affecting processes such as phagocytosis and migration [4,6].

In conclusion, Nhe1 is a critical regulator of directional migration in vegetative *Dictyostelium* cells and influences both chemotactic and electrotactic responses. By modulating intracellular pH, cytoskeletal organization, and key signaling pathways, such as PIP3 and calcium signaling, Nhe1 enables cells to effectively sense and respond to external directional cues. Future studies should investigate the precise molecular interactions between Nhe1 and other components of chemotactic and electrotactic signaling networks to provide deeper insights into the conserved mechanisms of directional migration.

## Supplementary Material

Supplemental Material

## Data Availability

The data that support the findings of this study are available from the corresponding author upon reasonable request.
