# Supplementary material for: Nhe1 is required for directional sensing in vegetative Dictyostelium cell migration
Source: Cell Adh Migr. 2025 Jun 3;19(1):2514374. doi: 10.1080/19336918.2025.2514374 (PMC12143694; doi:10.1080/19336918.2025.2514374)
Supplement: Supplemental Material [file KCAM_A_2514374_SM5651.docx]

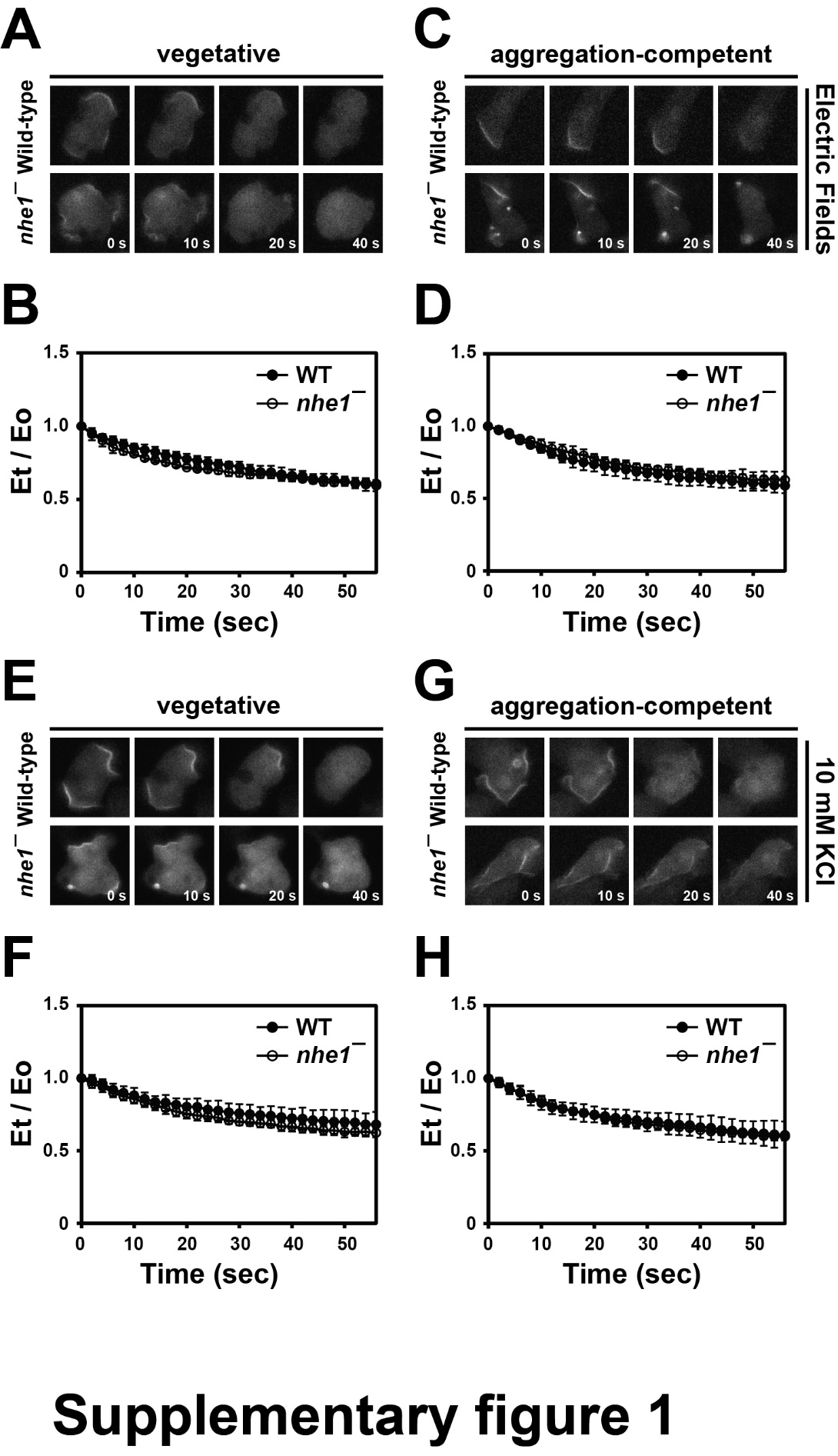


**Supplementary figure 1. Translocation of PH-GFP to the cell cortex upon EF and potassium stimuli**

(A-D) Translocation of PH-GFP to the cell cortex in response to EF. (A) Translocation of PH-GFP in vegetative cells upon EF stimulation. Vegetative wild-type and *nhe1* null cells expressing PH-GFP were uniformly stimulated with EF stimulation. Fluorescence images were captured at 2-s intervals for 1 min. Representative images at 0, 10, 20, and 40 s after EF stimulation are shown. (B) Translocation kinetics of PH-GFP to the cell cortex in vegetative cells upon EF stimulation. Fluorescence intensity at the cell cortex was quantified from time-lapse recordings. Graphs show the mean values from multiple cells in three separate experiments. Error bars indicate SEM. (C) Translocation of PH-GFP in aggregation-competent cells upon EF stimulation. Developed cells were prepared by pulsing with cAMP for 6 h. (D) Translocation kinetics of PH-GFP to the cell cortex in aggregation-competent cells upon EF stimulation. (E-H) Translocation of PH-GFP to the cell cortex in response to potassium stimulation. (E) Translocation of PH-GFP in vegetative cells upon potassium stimulation. Vegetative wild-type and *nhe1* null cells expressing PH-GFP were uniformly stimulated with potassium stimulation. The images were captured as described for EF stimulation. Representative images at 0, 10, 20, and 40 s after potassium stimulation are shown. (F) Translocation kinetics of PH-GFP to the cell cortex in vegetative cells upon potassium stimulation. Graphs show the mean values from multiple cells in three separate experiments. Error bars indicate SEM. (G) Translocation of PH-GFP in aggregation-competent cells upon potassium stimulation. (H) Translocation kinetics of PH-GFP in aggregation-competent cells upon potassium stimulation.

**Alt text:**

**Supplementary Figure 1. PH-GFP translocation in response to EF and potassium stimuli**

(A-D) PH-GFP translocation in vegetative (A, B) and developed (C, D) cells upon EF stimulation. Representative images and translocation kinetics shown. (E-H) PH-GFP translocation in vegetative (E, F) and developed (G, H) cells upon potassium stimulation. Data are mean ± SEM from three experiments.
